# Effectiveness of dual-task functional power training for preventing falls in older people: study protocol for a cluster randomised controlled trial

**DOI:** 10.1186/s13063-015-0652-y

**Published:** 2015-03-27

**Authors:** Robin M Daly, Rachel L Duckham, Jamie L Tait, Timo Rantalainen, Caryl A Nowson, Dennis R Taaffe, Kerrie Sanders, Keith D Hill, Dawson J Kidgell, Lucy Busija

**Affiliations:** Centre for Physical Activity and Nutrition Research, School of Exercise and Nutrition Sciences, Deakin University, Burwood, Victoria Australia; School of Medicine, University of Wollongong, Wollongong, New South Wales Australia; School of Human Movement Studies, University of Queensland, Brisbane, Queensland Australia; Edith Cowan University Health and Wellness Institute, Joondalup, Western Australia Australia; North West Academic Centre, Department of Medicine, University of Melbourne, Sunshine Hospital, St Albans, Victoria Australia; Institute for Health and Ageing, Australian Catholic University, Melbourne, Australia; School of Physiotherapy and Exercise Science, Curtin University, Perth, Western Australia Australia

**Keywords:** Dual-task training, Falls, Muscle power, Muscle function, Older persons, Cluster randomised controlled trial, Study protocol

## Abstract

**Background:**

Falls are a major public health concern with at least one third of people aged 65 years and over falling at least once per year, and half of these will fall repeatedly, which can lead to injury, pain, loss of function and independence, reduced quality of life and even death. Although the causes of falls are varied and complex, the age-related loss in muscle power has emerged as a useful predictor of disability and falls in older people. In this population, the requirements to produce explosive and rapid movements often occurs whilst simultaneously performing other attention-demanding cognitive or motor tasks, such as walking while talking or carrying an object. The primary aim of this study is to determine whether dual-task functional power training (DT-FPT) can reduce the rate of falls in community-dwelling older people.

**Methods/Design:**

The study design is an 18-month cluster randomised controlled trial in which 280 adults aged ≥65 years residing in retirement villages, who are at increased risk of falling, will be randomly allocated to: 1) an exercise programme involving DT-FPT, or 2) a usual care control group. The intervention is divided into 3 distinct phases: 6 months of supervised DT-FPT, a 6-month ‘step down’ maintenance programme, and a 6-month follow-up. The primary outcome will be the number of falls after 6, 12 and 18 months. Secondary outcomes will include: lower extremity muscle power and strength, grip strength, functional assessments of gait, reaction time and dynamic balance under single- and dual-task conditions, activities of daily living, quality of life, cognitive function and falls-related self-efficacy. We will also evaluate the cost-effectiveness of the programme for preventing falls.

**Discussion:**

The study offers a novel approach that may guide the development and implementation of future community-based falls prevention programmes that specifically focus on optimising muscle power and dual-task performance to reduce falls risk under ‘*real life*’ conditions in older adults. In addition, the ‘step down’ programme will provide new information about the efficacy of a less intensive maintenance programme for reducing the risk of falls over an extended period.

**Trial registration:**

Australian New Zealand Clinical Trials Registry: ACTRN12613001161718. Date registered 23 October 2013.

## Background

Falls in older adults are a common and major public health problem that can have serious consequences in terms of injury, pain, loss of function and independence, decreased quality of life and even death [1,2]. At least one third of community-dwelling people aged 65 and over fall at least once per year, and falls in these people are the leading cause of injury-related hospitalisation and death [3]. In Australia, it is projected that the total annual health cost attributable to fall-related injury will increase almost 3-fold to AUD$1.4 billion by 2051 [4]. Thus, there is a need to identify and implement cost-efficient and effective strategies to prevent falls in community-dwelling older adults to ensure that they live independently and relatively disease- and disability-free into old age.

It is well established that the causes of falling are varied and complex, but the age-related loss in muscle power has emerged as a major factor underlying impaired muscle function and disability in older people [5,6]. Muscle power reflects the ability of the muscle to produce force rapidly (that is, it is the product of force and velocity of contraction), and declines earlier and more rapidly with increasing age compared to muscle strength [7]. Many common daily functional tasks, such as the ability to get up from a chair, climb stairs and walk quickly to cross the road, are more strongly related to muscle power than muscle strength [8]. Indeed, the ability to recover from a loss of balance has been shown to be strongly related to the ability to step rapidly or grasp quickly for an object for support; factors associated with speed of generating force (power) [9]. The clinical relevance of this loss in muscle power has been demonstrated in a study that reported that older adults with low muscle power have a two- to three-fold greater risk of significant mobility impairment compared to individuals with low muscle strength [10]. There is also evidence that improvements in lower limb muscle power are more influential in producing clinically meaningful changes in muscle function than changes in muscle strength [11]. Thus, targeting deficits in lower extremity muscle power and movement velocity are likely to represent an effective strategy to optimise muscle function and reduce the risk of falls and related injuries in older people.

Extensive research into falls prevention has identified exercise to be an effective strategy to counteract key risk factors for falls, such as muscle weakness and poor balance, and reduce the risk of falling in older people. However, a recent meta-analysis of randomised controlled trials reported that exercise only reduced the risk of falling by an average of 16%, and that not all modes of exercise were equally effective with those studies that included walking being less effective [12]. While walking is a popular activity for many older adults and can substantially lower the risk of many chronic diseases, a large proportion of falls occur during walking [13]. Thus, there is a need to explore other novel exercise interventions that can improve multiple risk factors for falls (gait, balance, reaction time) and lower fall rates more effectively. Progressive resistance training (PRT) is one approach that is often recommended because of its reported benefits on muscle strength, but there are mixed findings with regard to the efficacy of this mode of training for preventing falls [12,14]. This is perhaps not surprising given the concept of training specificity. Most PRT programmes encourage slow velocity contractions (2 to 4 seconds concentric phase) at a moderate to high percentage of maximal force (approximately 60 to 80% of 1-repetition maximum strength (1-RM)). However, many common tasks related to mobility and daily perturbations require rapid coordinated and dynamic contractions within 50 to 200 ms, which is considerably less than the time needed to achieve maximal muscle force (approximately 400 to 600 ms) [15]. Thus, strategies that aim to enhance the ability to generate force quickly, and that are specific to tasks of daily living, are likely to be more relevant to the maintenance of muscle function and thereby prevent falls in older adults.

In recent years there has been interest in the role of high-velocity (HV) PRT, or power training, as a novel form of training to enhance muscle power and function in older adults. This mode of training is characterised by rapid concentric movements followed by a slower eccentric phase performed at moderate to high loads. In healthy young and older adults, a number of intervention trials have shown that HV-PRT using exercise machines conducted within a controlled setting was effective for improving muscle strength, power and functional performance [16-23]. The findings from a meta-analysis of the limited trials available also revealed that the functional gains following HV-PRT were greater than those that can be achieved through traditional PRT [24]. Most of the previous studies involving HV-PRT have used specialised exercise equipment within a controlled setting, which may not be readily accessible to many community-dwelling older adults. Importantly for older people, high-load or high-intensity training is not required as several studies have shown that training at low load and high velocity leads to similar (or even greater) gains in balance, movement speed and muscle strength compared to traditional slow speed PRT [23,25,26]. There is also evidence that dynamic functional exercises (stair climbing, chair stands, step-ups) performed rapidly and made progressively more challenging through the use of weighted vests or elastic bands/tubing can significantly improve muscle power and function [16,27]. However, a limitation of nearly all these studies is that they included small sample sizes and were performed in a controlled setting. To our knowledge, this will be the first community-based, randomised controlled trial to evaluate the long-term effects of HV-PRT on falls in older people.

For many older adults, the risk of falls is increased when they are required to undertake a secondary or concurrent cognitive or motor task (referred to as the ‘dual-task paradigm’), such as walking while talking, carrying objects or watching traffic [13,28,29]. Previous research has shown that cognitive deficits in older people, particularly deficits in executive function such as the ability to concentrate, to attend selectively, multi-task and to plan and strategise [30], are associated with both risk factors for falls (for example, postural instability, impaired gait, reduced ability to perform activities of daily living (ADL)); and future falls [31,32]. Indeed, there is evidence that under dual-task conditions, older adults exhibit poorer reaction times, reduced walking speed, increased sway, more frequent contact with obstacles whilst walking and slower step velocities, compared to single-task conditions [33-37]. Difficulties in dual-task conditions have also been associated with a history of falls and risk of future falls in community-dwelling older adults [31,37]. Several short-term trials and pilot studies in healthy older adults and those with stroke, Parkinson’s disease and dementia, have shown that balance or stepping programmes incorporating dual-tasking, such as exercising whilst performing a cognitive and/or motor task, were effective for improving balance and gait under dual-task conditions [38-45]. In contrast, single-task training was not transferable to balance performance under dual-task conditions [40]. This highlights the need for targeted falls prevention programmes that incorporate the principle of training specificity and replicate ‘real-life’ everyday situations in terms of how and where falls are likely to occur. To our knowledge, this study will be the first to investigate whether dual-task functional power training can reduce the rate of falls and improve risk factors for falls under single and dual-task conditions in older people at risk of falling. Furthermore, previous research has consistently shown that many of the benefits of exercise for falls prevention are lost following the cessation of supervised or intensive programmes [46,47]. Thus, there is a need to identify safe and effective strategies to maintain any initial benefits derived from structured or intensive programmes. In this study, we will examine the efficacy of a less intensive ‘step down’ maintenance programme on fall rates after an initial intensive 6-month structured training programme. We will also examine the cost-effectiveness of the intervention and maintenance programme for preventing falls in older community-dwellers.

The primary aim of this community-based, cluster randomised controlled trial is to evaluate whether dual-task functional power training (DT-FPT) can reduce the rate of falls in community-dwelling older adults at increased risk of falling. Secondary aims of the study are to:

1. Determine if DT-FPT can improve lower extremity muscle power, balance and gait under single and dual-task simulated ‘*real-life’* conditions.
2. Evaluate the effects of DT-FPT on health-related quality of life (HR-QoL), falls-related self-efficacy and cognitive function.
3. Evaluate the efficacy of a ‘step down’ maintenance programme on falls risk.
4. Determine the cost-effectiveness of DT-FPT and the ‘step down’ maintenance programme for preventing falls. The results will be expressed as the incremental cost of the intervention in monetary terms per unit gain per fall averted.

## Methods/Design

### Study design

This is an 18-month, community-based, cluster randomised controlled trial in which older adults residing in retirement villages, who are at increased risk of falling, will be randomly allocated to: 1) an exercise programme involving dual-task functional power training (DT-FPT), or 2) a usual care control group. The intervention is divided into 3 distinct phases: 6 months of supervised and structured DT-FPT, a 6-month ‘step down’ maintenance programme, and a 6-month follow-up. Multi-care level retirement villages will be recruited but only people that live independently in apartments or units, but share common-room facilities, will be recruited. The trial is managed by the Centre for Physical Activity and Nutrition Research at Deakin University, Burwood, Melbourne, Australia and is funded by a National Health and Medical Research Council (NHMRC) Project Grant (ID1046267). The study has been approved by the Deakin University Human Research Ethics Committee (HREC 2013-051), and is registered with the Australian and New Zealand Clinical Trials Registry (ACTRN12613001161718). Written informed consent will be obtained from all participants prior to commencement of the trial.

### Participants

A total of 280 men and women aged 65 years and over at an increased risk of falling (see below and Table 1) and who currently reside in retirement villages will be invited to participate in this study.

**Table 1:** **Falls and fracture risk questionnaire for inclusion into the trial**

**Risk factor**	**Guidelines**	**Score (circle)**
History of falling^a^	Self-reported risk of falling (1 or more falls in past year)^a^	3
Age	>75 years	2
	70 to 75 years	1
Low trauma fracture^b^ or osteoporosis	Since age of 50 years (T-score < −2.5 SD at the hip or spine)	2
Difficulty when rising from a chair or toilet without using arms	When getting up from a chair or the toilet do you use your arms?	2
History of slipping or tripping	Have you had a slip or trip in the past year?	2
Medication use	How many medications are you currently taking? If four or more include as two points	2
Use of walking aid	Yes or No?	2
One psychoactive drug	Do you take any medications to treat anxiety, panic attacks or insomnia seizures?	1
On feet < 4 hours per day	Are you on your feet < 4 hours a day?	1
Multi-focal glasses	Do you wear multi-focal glasses?	1
Poor vision (for example, cataract, glaucoma)	Self-reported or assessed by primary care physician - Do you have cataract or glaucoma?	1
When walking	Do you ever have trouble walking or feeling unsteady on your feet?	1
Self-rated health as fair or worse compared to last year	Excellent/Good/Fair/Poor/Very poor (very poor = 1 point)	1
Thinness	Body mass index (BMI) < 20	1
High risk of vitamin D deficiency	In summer, ‘Do you spend < 10 minutes per day outdoors (with part of your body exposed to sunlight), without taking vitamin D supplements between the hours of 10am to 3 pm’?	1
	OR	
	In winter, ‘Do you spend < 30 minutes per day outdoors (with part of your body exposed to sunlight), without taking vitamin D supplements’?	
Total score (Include if score ≥ 3)**:**		

^a^A fall is defined as an event that results in unintentionally coming to rest on the ground or a lower surface, other than as a consequence of a sudden onset of paralysis, epileptic seizure, or overwhelming external force.

^b^A low trauma fracture is defined as a fragility fracture of the spine, hip or wrist.

#### Recruitment

Retirement villages within the Melbourne metropolitan region and surrounding areas in Victoria, Australia will be approached via mail or telephone and invited to participate in the study. For those that agree and consent to participate, the manager will be asked to provide a single numbered list of residents aged 65 years and over who will receive an invitation letter to participate in the study. Advertisements will also be placed in village newsletters and on relevant notice boards, and information sessions will be conducted by the research staff at each village. While the total number of villages recruited will depend on the size of each village (number of residents), the goal is to recruit between 15 to 20 people per village. All participants who express an interest in the study will be required to undergo screening to determine their eligibility to participate in the trial as outlined below. To maximise recruitment, permission will also be sought from each retirement village to invite non-residents aged 65 years and over who fulfil the inclusion criteria to participate in the trial. These participants will be recruited via doctor/health professional referrals, a local media campaign and advertisements placed on relevant notice boards and word of mouth.

#### Screening and eligibility

All interested participants will be screened over the telephone and will be eligible for the study if they score ≥ 3 points on an algorithm adapted from identified risk factors for falls (Table 1). In addition, participants must be able to speak English proficiently, walk unaided or with minimal assistance (walking stick or walker) for at least 50 meters and be cognitively intact (score ≤ 2 errors on the Short Portable Mental State questionnaire) [48]. All eligible participants will then be screened with the Exercise and Sports Science Australia (ESSA) exercise screening tool to evaluate any contraindicated medical conditions to exercise. Participants answering ‘yes’ to any of these screening questions will be required to obtain medical clearance from their local doctor prior to participating in the intervention. For all other participants, an information letter outlining the study aims and its requirements will be sent to their doctor to inform them that their patient is participating in this research trial.

Participants will be ineligible based on the following criteria: 1) current or prior participation in a structured progressive resistance training (PRT) programme and/or organised balance training more than once per week in the past 3 months; 2) acute or terminal illness likely to compromise exercise participation; 3) unstable or ongoing cardiovascular/respiratory disorders; 4) musculoskeletal or neurological diseases disrupting voluntary movement or that might limit training; 5) upper or lower extremity fracture in the past 3 months, or 6) visual impairment not corrected with glasses. To assess for possible response bias, data on the age and sex for all eligible non-participants residing at each retirement village where the study will be conducted will be collected.

#### Randomisation

**Figure 1:**
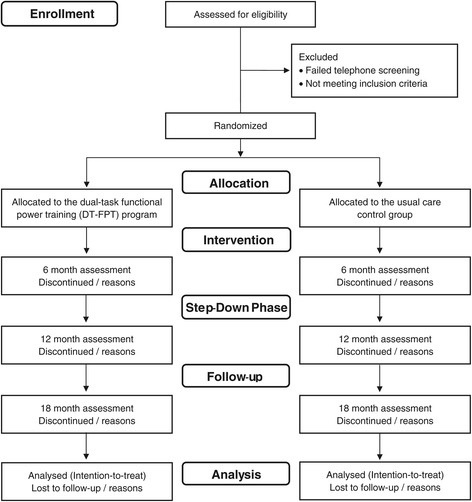
**Flow diagram of the progress from screening to the final follow**-**up assessment.**

Randomisation will be performed by cluster (village) to avoid any potential contamination and facilitate logistical arrangement. Each village will be given an ID number and group assignment by blocks of 2, stratified by village size (<75 or ≥ 75 residents), will be performed by a researcher not involved in the study using computer-generated random numbers (Microsoft Excel, Microsoft Corp., Redmond, WA, USA). A flow diagram of the study protocol is outlined in Figure 1.

#### Allocation concealment and blinding

Retirement villages will be allocated to the intervention or usual care only after all participants have completed baseline testing. The retirement villages, the residents and the research staff undertaking the baseline testing will be blinded to the allocation until this point. It is difficult for the participants within the exercise intervention to be blind in trials of exercise, as well as for all research staff to be blind to the allocation of the participants as they will often recruit participants and undertake baseline and follow-up assessments. Thus, for pragmatic reasons not all research staff will be blinded in this study. However, research staff undertaking the baseline and follow-up assessments and the statistical analysis will be blinded to the treatment allocation of the retirement villages.

### Intervention

#### Dual-task functional power training

Participants residing in the villages assigned to the DT-FPT programme will be asked to train twice a week for 26 weeks. All training will be conducted on-site at each retirement village in small groups (8 to 10 per group), and will be implemented and supervised by accredited exercise physiologists (AEPs), physiotherapists or experienced Certificate IV personal trainers. All trainers involved in the study will be required to attend a ‘train-the-trainers’ workshop and will be provided with a detailed instructor manual that will contain all the necessary information to implement the exercise programme. The delivery of the programme will be standardised through regular quality assurance visits and monthly trainer meetings with the research staff.

The 26-week programme will be divided into an initial 2-week familiarisation (orientation) period, followed by 3 distinct but interrelated 8-week phases termed ‘mesocycles’. Each mesocycle will be further divided into 2-week achievable microcycles that are designed to be progressively more challenging. Each training session is designed to be completed within 45 to 60 minutes and will be divided into 4 components: 1) a warm-up consisting of rhythmic and range of motion exercises, 2) challenging balance and mobility activities, 3) HV-FPT, and 4) a cool-down. Dual-task activities will be incorporated into the challenging balance/mobility and HV-FPT. All programmes will be individualised to the functional capabilities of each participant based on their initial assessment.

For the challenging balance and mobility training, participants will be asked to engage in a range of activities designed to simulate common daily functional tasks such as avoiding and stepping over obstacles, weight-shifts and leaning to reach objects. Particular emphasis will be placed on training the lower limb muscles (ankle dorsi- and plantar-flexors, knee flexors and extensors, hip abductors, adductors and extensors) that are associated with balance and mobility. The programme will become progressively more challenging through the inclusion of more difficult postures with a gradual reduction in the base of support, dynamic movements that increasingly perturb the centre of gravity, reducing sensory input, negotiating different environmental hazards and the use of dual-task activities. For each session, participants will be required to perform at least two challenging balance or mobility exercises.

For the HV-FPT component, the goal will be to increase lower limb muscle power, movement speed, force and mobility. Participants will be instructed to perform a series of resistance and functional tasks where all repetitions for each concentric (shortening) phase of the exercise will be performed as quickly as possible, while the eccentric (lengthening) phase of the exercise action will be controlled over 3 to 4 seconds. The exercises will become progressively more challenging by increasing the resistance, difficulty or changing the exercise to another more complex task. Core exercises will include: multi-directional rapid stepping and bench stepping, combined with functionally relevant resistance exercises (squats, hip abduction, hip extension and ankle plantar- and dorsi-flexion). Weighted vests, thera-bands, tubing, hand-held weights and sand bags will be used to increase the training loads. Participants will perform 2 sets of 10 to 20 repetitions progressing to 50 repetitions for the stepping exercises. For the functional resistance exercises, participants will perform 2 sets of 10 to 15 repetitions at an intensity of 4 to 6 (moderate to hard) on the 10-point Borg’s Rating of Perceived Exertion scale [49], and resistance will be increased as tolerated using this scale.

The dual-task training will incorporate a combination of cognitive and secondary motor tasks that will be performed simultaneously whilst undertaking the challenging balance/mobility and HV-FPT. Prior to incorporating the secondary tasks into the programme, participants will undertake at least 2 weeks of training without this component to ensure safety and confidence with the single-task exercises. Cognitive and motor tasks will then be progressively incorporated into the programme. Examples of cognitive tasks that may be incorporated into the programme include: tasks related to verbal fluency (for example, listing words within a category (names of animals, vegetables, fruit, and so on) or by letter (a word that begins with ‘D’)), serial subtraction (for example, subtracting three from a given start number) and information processing (for example, stepping rapidly in a given sequence on command or from memory) onto thin slip-resistant mats or discs that may be different in colour. Dual tasks will be progressed or changed on an individual basis every 4 weeks or as needed.

To maximise social interaction and enjoyment and enhance adoption and maintenance, participants will train in small groups (8 to 10 per group) and we will incorporate established behavioural models that have previously been successful in older adults. Effective strategies have included: social support (buddy system); regular feedback (fitness testing); positive reinforcement through rewards (T-shirts, caps) and public recognition in newsletters for attendance and adherence; and ongoing education via fact sheets and newsletters.

#### Usual care control group

Participants residing in the villages assigned to the control group will receive their usual care from their medical practitioner and community services. They will also be encouraged to adhere to the current guidelines of at least 150 minutes per week of moderate intensity physical activity. They will also receive standardised falls prevention and physical activity advice in the form of booklets (Department of Health: ‘*Don’t fall for it - falls can be prevented’*, and *‘An active way to better health’*) along with generic project newsletters that contain project updates to help minimise attrition over the 18-month duration. There is no evidence that the provision of written falls prevention or physical activity advice alone reduces falls risk [50].

#### ‘Step down’ maintenance programme and follow-up

A 6-month ‘step down’ maintenance programme will be introduced after the supervised training for the intervention group since previous trials have shown that the benefits of exercise on falls incidence are largely lost after programme cessation [46,47]. The ‘step down’ represents a decrease in the level of free supervised training that will be offered to the participants by the exercise staff who delivered the first 6-months of the programme. Prior to this phase we will liaise with all village managers to discuss strategies as to how they can continue to offer the programme within their village without the intensive intervention of the research staff. Village managers will be provided with a number of potential options as to how they can continue to offer the programme to the residents. To facilitate this transition, participants will be offered 1 weekly 1-hour exercise class led by our trained exercise staff who have completed our ‘train-the-trainers’ workshop. In addition, since one of the goals of the main intervention is to improve gait and muscle function under dual-task conditions, participants will also be encouraged to undertake at least one brisk walking session per week. Within each village, staff/participants will be encouraged to form walking groups. Compliance with the ‘step down’ programme will be monitored by attendance sheets completed by the staff at each village and a diary for recording the walking. After the 6-month ‘step down’ programme, participants will be followed for a further 6 months to establish the long-term residual effects on falls. Participants and staff at the villages will not receive any support or advice during this final 6-month follow-up.

### Outcome measures

A summary of the outcome measures is shown in Table 2. Participants will attend 4 testing sessions at their retirement village throughout the study (baseline, 6, 12, and 18 months).

**Table 2:** **Summary of the outcome measures**

**Outcome measures**	**Data collection method**	**Data collection points**			
		**Baseline**	**6 months**	**12 months**	**18 months**
Primary outcome measure					
Falls	Monthly falls calendars	Monthly			
Secondary outcome measures					
Muscle power	5-consective sit-to-stands	x	x	x	x
Muscle strength	30-second Sit-to-Stand test	x	x	x	x
	Isometric knee extensor strength	x	x	x	x
	Isometric dorsi-flexion strength	x	x	x	x
	Hand grip dynamometer	x	x	x	x
Muscle function and balance	Timed up-and-Go: single- and dual-task	x	x	x	x
	Four-squared step test	x	x	x	x
	Choice reaction time	x	x	x	x
Single- and dual-task gait	Gait (4.9 m electronic walkway): gait velocity, cadence, step length, double support time, stride wide and dual-task cost	x	x	x	x
Instrumental Activities of Daily	Lawton IADL questionnaire	x	x	x	x
Living (IADL)					
Health-related Quality of Life	Short Form (36) version 2 questionnaire and the Assessment of Quality of Life - 6D scale	x	x	x	x
Cognitive function	CogState Brief Battery computerised tests	x	x	x	x
Falls self-efficacy	Falls Efficacy Scale-International questionnaire	x	x	x	X
Additional measures					
Anthropometry	Height, weight and body mass index	x	x	x	x
Body composition	Bioelectrical impedance (fat mass, fat-free mass and % fat)	x	x	x	x
Physical activity	Community Healthy Activities Model Programme for Seniors questionnaire	x	x	x	x
Falls-related injuries	Monthly calendar	Monthly			
Health and medical history	Lifestyle questionnaire	x	x	x	x
Dietary intake	Anti-Cancer Council Food Frequency Questionnaire	x	x	x	x
Adverse events^a^	Questionnaire and interview		x	x	x
Exercise programme compliance	Calculated from exercise cards	Collected every month			

^a^Adverse events will also be collected at 3 months.

### Primary outcome measures

The primary outcome will be the number of falls over the 6-, 12-, and 18-month period. The initial comparison of falls outcome between the two groups will be analysed according to the number of falls per person year and the proportion sustaining one or more falls. Subsequent analyses will allow for the non-independence of multiple events for the same participants. Participants will record all falls ((F (fall) or N (no fall)) each day on a monthly falls and adverse events calendar, which will be returned monthly via postage-paid mail. Any participants who do not return the monthly falls calendar will be contacted by telephone by the research staff. When a fall is recorded, a research staff member will administer a standardised questionnaire via telephone to record more specific details of the fall location, cause, injury, treatment and the healthcare utilisation. The fall event data will be coded using the International Classification of Diseases, 10th edition (ICD-10).

### Secondary outcome measures

The secondary outcome measures to be assessed at baseline, 6, 12, and 18 months (unless stated) will include: changes in lower limb functional muscle strength and power, isometric knee extensor, dorsi-flexor and hand grip strength, dynamic balance and reaction time, gait, Instrumental Activities of Daily Living (IADL), quality of life, cognitive function and falls- related self-efficacy (Table 2).

#### Functional muscle strength and power

Functional muscle strength of the lower extremities will be assessed using the 30-second Sit-to-Stand (STS) test. Participants will start from a seated position in a chair, with arms folded across the chest, and will be instructed to stand fully upright and return to the seated position as many times as possible in 30 seconds. The final score will be the number of complete stands recorded during this time. The STS test has been shown to have a good test-retest reliability correlation of 0.84 to 0.92 in a sample of community-dwelling men and women over the age of 60 years [51].

Functional lower limb muscle power will be assessed using five consecutive sit-to-stands [52]. Participants will be fitted with a tri-axial accelerometer (x-BIMU Bluetooth Kit, x-io Technologies Limited, Ascot, UK, gyroscope ±2,000°/s, accelerometer ±16 g, 16-bit A/D conversion, sampled at 256 Hz) at the right hip, and will be instructed to perform each STS as rapidly as possible. For each complete STS, the mean concentric power relative to body weight (W/kg) will be calculated based on the product of acceleration and velocity in line with previous research [52].

#### Isometric muscle strength

Bi-lateral maximal isometric knee extensor strength will be measured using Lord’s strap assembly incorporating a strain gauge (Neuroscience Research Australia, Sydney, Australia). Briefly, participants will be seated with their hip and knee at an angle of 90°, with a strain gauge attached to a strap around the leg about 10 cm above the ankle joint. A strap will also be placed around the thighs to prevent movement of the pelvis and minimise any contribution of the gluteal muscles. Participants will be asked to perform 2 practice trials prior to the completion of 2 maximal efforts with a 60-second rest between each test. For analysis, the maximal knee extensor strength (kg) of each leg will be expressed per unit of lower leg length to compensate for the length of the lever arm. This test has been shown to have excellent test-retest reliability (Pearson’s r = 0.92) [53].

Bi-lateral maximal isometric dorsi-flexion strength will be assessed using a dorsi-flexion dynamometer (Neuroscience Research Australia, Sydney, Australia). Participants will be instructed to sit on a 45-cm high chair with the foot strapped securely to a spring-gauged plate attached to a strain gauge load cell. Participants will be instructed to perform 1 practice trial followed by 2 maximal effort muscle contractions, interspersed by a 10- to 15-second rest [54]. For analysis, maximal dorsi-flexion strength (kg) of each leg will be recorded.

Bi-lateral maximal isometric grip strength will be assessed using a hand-held dynamometer (Jamar dynamometer, Asimov Engineering Co., Los Angeles, CA, USA). Participants will be instructed to sit on a standard height chair with their shoulder adducted and neutrally rotated, elbow flexed at 90°, forearm neutral and hand slightly extended. They will be instructed to perform 1 practice trial, followed by 2 maximal effort muscle contractions by squeezing the handle of the dynamometer as forcefully as possible. For analysis, maximal grip strength (kg) of each hand will be recorded.

#### Muscle function and performance

The Four-square step test (FSST), Timed up-and-go test (TUG), and choice stepping reaction time (CSRT) will be used to assess lower limb muscle function. Each of these tests will be performed in the participant’s own footwear.

The FSST provides a measure of dynamic balance and stepping speed in four directions [55]. Participants will be instructed to step forward, sideways, and backwards over four canes resting flat on the floor in a cross formation. The test begins with the participant moving first in a clockwise direction and returning in a counter-clockwise direction to the start square. Participants will be instructed to complete the task as quickly as possible without touching or stepping on the canes and, if possible, to face forward during the entire sequence. They will also be instructed to ensure that both feet make contact with the floor in each square. After 1 practice trial, participants will complete the test and the time (in seconds) taken to complete the sequence will be measured with a stopwatch and recorded as the final score. The FSST has been shown to discriminate between community-dwelling older adults with and without a history of falls [55]. The test has an 89% multiple falls sensitivity and an 85% specificity for non-fallers with a cut off score of greater than 15 seconds [55]. The test has been shown to have a high test-retest reliability of 0.98 [55].

The TUG test is a measure of dynamic balance using three commonly performed functional activities of daily living: standing and sitting, walking, and turning [56]. Participants will be seated in a chair (height 45 cm) that will be placed at the end of a marked 3-meter walkway. On the command ‘go’, participants will be instructed to stand up, walk at a comfortable speed for 3 meters, turn back to the chair and sit down. The test will be performed under both single- and dual-task conditions. The dual-task condition will involve participants completing the task while counting backwards in threes from a randomly selected number. All participants will be given a practice trial and one test run. Participants who require a usual indoor walking aid (cane or walker) will perform the test with the use of their aid. A stopwatch will be used to record the time taken (in seconds) to complete each test. Prior to completing the dual-task TUG test, the participants cognitive ability while seated will be assessed by asking them to count backwards in threes from a random number. The time taken to complete 10 subtractions from this random number will be recorded. The number of errors that occur during the seated and dual-tasking test will be recorded for all participants. ‘Dual-task cost’, which represents the change in performance with the addition of a second task, will be calculated as:

$$ \mathrm{Dual}\hbox{-} \mathrm{task}\ \mathrm{TUG}\ \mathrm{performance}\ \hbox{-}\ \mathrm{single}\ \mathrm{TUG}\ \mathrm{task}\ \mathrm{performance}/\mathrm{Single}\ \mathrm{TUG}\ \mathrm{task}\ \mathrm{performance} \times 100 $$

The TUG test has an interrater reliability of 0.99 and an 87% prediction rate for identifying fallers and non-fallers when performed with a cognitive dual task [57].

Choice stepping reaction time (CSRT) is a measure of a participant’s ability to step as quickly as possibly onto one of four foot panels [58]. This test has been shown to be an independent predictor of falls risk [58]. Participants will stand on a non-slip choice reaction mat (0.8 × 0.8 m) that contains 4 rectangular panels (32 × 13 cm), 1 in front of each foot and 1 to the side of each foot [58]. One panel per trial will illuminate in a random order, and participants will be instructed to step onto the illuminated panel as quickly as possible, using the left foot only for the two left panels (front and side) and the right foot only for the two right panels. Following a practice trial, participants will complete a single trial that will involve 12 target stepping actions in which 12 green arrows will appear in a random sequence. Following this trial, participants will complete the same activity but with the addition of a dual task. For this trial, participants will be asked to step when a green arrow appears and to not step when a purple arrow appears. A total of eight green panels and four purple panels will be displayed in a random sequence. CSRT will be measured as the time period between the illumination of an arrow and the foot making contact with it, and the average reaction time (in ms) will be recorded. In addition, the total time will be subdivided into: 1) the reaction time measured from the illumination of the arrow to movement initiation (lift off), and 2) the movement time measured from movement initiation to foot contact with the arrow/mat (‘step down’).

#### Functional gait

Gait function will be assessed using the ProtoKinetics Zeno system (ZenoMetrics LLC, Peekskill, NY, USA), which comprises a computerised walkway with the ProtoKinetics Movement Analysis Software (PKMAS) programme. The ProtoKinetics Zeno walkway system is a 2-foot wide by 16-foot long mat that contains a 16-level pressure sensing pad with 18,432 pressure sensors arranged in a grid pattern with a spatial resolution of 0.5 cm and a sampling frequency of 120 Hz. Participants will be asked to perform 18 trials on the Zeno system, wearing comfortable footwear, at their preferred walking speed, and with their usual indoor walking aid if required. Three different gait conditions will be assessed: 1) simple walking; 2) walking while counting backwards in 7s from a computerised derived random number between 70 and 99 (cognitive dual task), and 3) walking while carrying a tray whilst balancing a ball in a target (motor dual task). For the cognitive and motor dual task, the number of errors in counting backwards and the number of times the ball deviates from the centre of the target will be recorded. Motor dual-task trials will not be completed by those participants requiring an indoor walking aid. For each walking trial, all participants will start standing 1 m behind the walkway, and on the command ‘go’ they will walk the length of the Zeno mat to a cone that will be placed 1 m past the end of the walkway. All participants will be given standardised verbal instruction followed by one practice trial for each walking condition. Participants will not be given any instruction to prioritise attention to either the dual task or walking. The following gait parameters will be calculated using the mean of the three trials: gait velocity (m/s), cadence (steps/minute), step length (cm), double support time (seconds) and stride width (cm). ‘Dual-task cost’, which represents the change in performance with the addition of a second task, will be calculated as:

$$ \mathrm{Dual}\hbox{-} \mathrm{task}\ \hbox{-}\ \mathrm{single}\hbox{-} \mathrm{task}\ \mathrm{walking}/\mathrm{Single}\hbox{-} \mathrm{task}\ \mathrm{walking} \times 100 $$

In addition, we will quantify each participant’s cognitive and motor ability whilst not undertaking a secondary task. Each participant will be asked to count backwards aloud by 7s while standing still. Once ten subtractions have been completed, the time taken and the number of errors will be recorded. Similarly, each participant will be asked to balance the ball in the centre of the tray for 20 seconds while standing still. The number of times the balls deviates from the centre of the target will be recorded. The order of the single and dual-task conditions will be randomised across participants.

#### Instrumental Activities of Daily Living (IADL)

Instrumental Activities of Daily Living (IADL) will be assessed using the Lawton IADL questionnaire [59], which is widely used to assess independent living skills. The tester rates the participant across eight functional abilities, including use of the telephone, shopping, food preparation, housekeeping, laundry, mode of transport, taking medications and handling finances. Participants are scored according to their highest level of functioning for each category. A summary score ranges from 0 (low function, dependent) to 8 (high function, independent).

#### Health-related quality of life (HR-QoL)

The Short Form 36 version 2 (SF-36 v2) questionnaire will be used to measure health-related quality of life (HR-QoL) [60]. The SF-36 v2 questionnaire consists of 36 items that cover 8 domains of HR-QoL: Physical functioning, Role-Physical, Bodily pain, General health, Vitality, Social functioning, Role-Emotional and Mental health [60]. The SF-36 survey has been established as valid and reliable in both interview and survey formats, and shows high reliability (α = 0.77 to 0.92) in people aged 65 years and older [61,62]. The scores for the SF-36 were originally based on a 0 to 100 scale, with higher scores indicating a better quality of life. However, this study will report Australian norm-based scores according to previously published guidelines [63]. The use of norm-based weights gives each domain score a mean of 50 and a standard deviation (SD) of 10, allowing change in scores to be assessed on a comparable scale. Basing the weights on Australian data (from the 2004 South Australian Health Omnibus Survey) [63] helps to account for cultural differences between populations in the way in which health and QoL are viewed [63]. Summary measures of the physical and mental components of the survey will also be calculated based on a factor analysis of the 8 domains among participants in the 2004 South Australian Health Omnibus Survey, yielding two separate overall summary scores: the physical component summary (PCS) and the mental component summary (MCS) scores.

Participants will also be asked to complete the Assessment of Quality of Life - 6D scale (AQoL-6D) questionnaire, which is a 20-item self-report instrument that considers six dimensions of health, including independent living, relationships, mental health, coping, pain and senses [64].

#### Cognitive function

The CogState Brief Battery computerised test (http://cogstate.com/) will be used to assess cognitive function. More specific details about these tests have been described previously [65,66]. Briefly, the tests have been designed to be easily administered and repeatable without eliciting practice or learned effects, and have been shown to provide sensitive and valid measurement for a range of different cognitive functions [65,66]. The battery of five tests that will be used for this study include: Groton maze learning test (measure of executive function and spatial problem solving), the Detection task (psychomotor function and speed of processing), the Identification task (visual attention and choice reaction time), One Card Learning task (visual learning with a pattern separation model) and the One Back task (working memory/attention).

#### Falls efficacy

Falls efficacy will be measured using the 16-item Falls Efficacy Scale-International (FES-I) version questionnaire [67], which is a tool for measuring the level of concern about falling during social and physical activities indoors and outdoors on a 4-point Likert scale (1 = not at all concerned to 4 = very concerned).

### Additional measures

#### Anthropometry and body composition

Height will be measured with a standardised portable stadiometer to the nearest 0.1 cm. Body mass will be measured to the nearest 0.1 kg. Body composition will be assessed using a whole body bioelectrical impedance (BIA) segmental body composition scales (TANITA BC-418, Tanita, Tokyo, Japan). This is a single-frequency (50Hz) BIA device that uses 8 polar electrodes that can provide a measure of whole body and segmental (arms and legs) fat mass (FM) and fat-free mass (FFM). An algorithm incorporating impedance, age and height will be used to estimate percentage fat mass. To standardise the measurement of weight and body composition, participants will be instructed to refrain from eating a meal 1 to 2 hours prior to the testing and to ensure normal hydration status. Participants will be measured wearing light clothing, standing erect and barefoot on the analyser’s footpads.

#### Lifestyle and medical history

A lifestyle questionnaire will be used to obtain detailed information on the participant’s ethnic and education background, employment history/status, medical history, previous history of any falls and fractures, family history of osteoporosis, current medication and dietary supplement use, smoking status, weekly television viewing and sitting time, and sun exposure habits.

#### Diet

Dietary intake will be assessed at each testing assessment using the Dietary Questionnaire for Epidemiological Studies Version 2 (DQES v2), a modification of the Cancer Council Food Frequency Questionnaire (FFQ) [68]. The DQES v2 covers 5 main types of dietary intake: 1) cereal foods, sweets and snacks; 2) dairy products, meats and fish; 3) fruit, 4) vegetables and 5) alcoholic beverages, incorporating 80 items. The DQES v2 is designed to be self-administered to determine the usual eating habits of the participants over the past 12 months at baseline. Participants will be instructed to record how often they ate each food listed on average over each time period. All questionnaires will be checked by the researchers for completeness. The performance of the Cancer Council FFQ has been evaluated in studies comparing the results of the questionnaire with those from weighed food records showing good agreement between methods [69].

#### Habitual physical activity

Total leisure and recreational physical activity will be assessed using the Community Healthy Activities Model Programme for Seniors (CHAMPS) questionnaire. This questionnaire has been designed for use in older adults and has been shown to be reliable, valid and sensitive to the changes in physical activity behaviour [70]. At each assessment, participants will record their weekly frequency and duration of participation in a ‘typical week’ of the preceding 4 weeks. The results will be reported as estimated kilojoules per week spent in moderate- to high-intensity activities.

#### Exercise compliance

Compliance with the exercise programme will be assessed by attendance at the supervised exercise sessions and completion of personal exercise cards that will be completed by the participants and checked by the trainers after each session.

#### Adverse events

All adverse events will be self-reported by the participant at 3, 6, 12 and 18 months and assessed by the research staff for seriousness, expectedness and causality following the guidelines recommended by the National Institute for Ageing (NIA) (http://www.nia.nih.gov/research/dgcg/clinical-research-study-investigators-toolbox/adverse-events). For this study, an adverse event will be defined as any health-related unfavourable or unintended medical occurrence (sign, symptom, syndrome, illness) that develops or worsens during the period of observation in the trial. Adverse events will be closely monitored until a resolution or stabilisation is achieved, or until it has been shown that the study intervention is not the cause of the injury. Participants will be asked to contact the research staff immediately in the event of a serious adverse event. Any adverse event sustained during the exercise programme will be recorded by the trainers and immediately reported to the research staff. The chief investigator will be informed and shall determine the seriousness and causality in conjunction with any medical staff treating the event. A serious adverse event that is deemed related to or suspected to be related to the exercise intervention will be reported to the ethics committee.

#### Cost-effectiveness analysis (CEA)

The CEA will be used to determine the monetary treatment costs of implementing the exercise programme and cost of outcomes (for example, falls and their consequences). The results will provide an incremental cost per fall averted for the DT-FPT and ‘step down’ programme. The incremental cost-effectiveness ratio (ICER) is the ratio of the incremental difference in treatment cost to the cost-saving (fewer falls). The ICER will be used in future decision-making on the allocation of resources, which maximises the health effects for a given amount of resources. The following measures will be collected: 1) the costs of implementing the programme (for example, staff costs, training, vehicle costs, capital costs and consumables), and 2) costs of health services (for example, directly related to a fall including inpatient hospital admissions, emergency department presentations and other health and community service contact) derived from the monthly calendars. The results will be expressed as the incremental cost (dollars) per fall averted among those randomised to the DT-FPT and ‘step down’ programme versus usual care. Research-related costs will not be included in the CEA.

### Sample size

Based on previous research in Australia [71-73], we anticipate that approximately 45% of the usual care control group will experience a fall throughout the study. To detect a 40% reduction in the rate of falls (for example, from 45% to 27%) in the DT-FPT group, we estimate that we will require 118 participants per group (2-tailed, *P* < 0.05 and power of 0.8). This allows for a 15% loss to follow-up due to death or withdrawal from the study. To account for cluster randomisation, we will assume a conservative intra-cluster correlation coefficient of 0.01 [74], giving a design effect of 1.19, assuming approximately 15 to 20 participants are recruited per village. This gives a sample size of 140 per group (280 participants from approximately 15 villages). This sample size will also provide sufficient power (0.9, 2-tailed, *P* < 0.05) to detect significant and clinically meaningful differences in many of the secondary outcome measures. Previous research has shown that 6 months of high-velocity resistance training in older adults can result in a 50% increase in peak muscle power (SD 18%) [20]. This mode of training has also resulted in a reduction in chair rise time of approximately 11% (SD 12%) and stair climbing time of approximately 7% (SD 10%) [20,21]. For the usual care control group, we estimate a mean change of 0 (SD 10%). Thus, to achieve 90% power at *P* < 0.05 (2-tailed), we estimate that 46 participants per group would be required to demonstrate between group differences of this magnitude in these measures. We will also have sufficient numbers to detect the smallest clinically significant differences in other measures of function and the SF-36. Perera *et al*. [75] calculated that a small meaningful change (difference) in function is around 5% (0.05 m/s gait speed) and a substantial change is around 10% (0.10 m/s gait speed). Since we will recruit a cohort at increased risk of falling, we anticipate greater changes based on previous work in healthy community-dwelling older adults (gait speed +7%, chair rise +13%) [21]. We estimate that 32 to 42 participants per group will be needed for a statistical power of 0.9 (2-tailed, *P* < 0.05) to detect differences between the groups of 14 to 25% in gait speed and chair rising time. Using data from a pilot study on dual-task stepping exercise [45], we estimate that we will need 48 participants per group to detect a 20% difference (SD 25%) in dual-task cost at 90% power (2-tailed, *P* < 0.05). For the sub-scales of the SF-36, we anticipate a change of 10 points in the DT + FPT groups with a small age-related change (decrease) in the controls. In various patient groups a 5 to 10% change is regarded as the minimal important difference. We note that the effects of resistance training on SF-36 are mixed, but differences of 15 to 52 points for the various sub-scales have been reported [76].

### Statistical methods

Primary analyses will be conducted on an intention-to-treat basis using STATA statistical software release (STATA, College Station, TX, USA). Per protocol analysis will also be performed by including all participants who are at least 80% compliant to the exercise (as measured by the number of exercise sessions attended). Initially, descriptive statistics will be computed to compare the intervention and control groups on background variables and baseline measures. Imbalances on prognostic factors between the groups will be adjusted for during analyses. Calculation of QoL scores (SF-36 v2, AQol-6D) will utilise published ‘weightings’ most relevant to this population. Baseline measures and changes in outcome variables over the study period for each study arm will be presented as means (± SD) with 95% confidence intervals. The effect of the intervention on the primary outcome variables will be assessed using negative binomial regression, to account for the non-independence of multiple events for the same participants and to allow for over-dispersion [77]. To account for clustering, the negative binomial regression analysis will be carried out using the generalised linear mixed modelling approach, with observations clustered within retirement villages; village will be modelled as a random effect. Differences between the intervention and control groups on the secondary outcome measures will be examined using linear mixed models with assessment times clustered within individuals and individuals clustered within villages. Village and, where appropriate (as determined by the likelihood ratio tests) individuals, will be modelled as random effects. The secondary outcome variables will be checked for normality prior to analysis and transformed appropriately if necessary. For both primary and secondary outcomes, random effects will be computed utilising robust standard errors. For all outcomes, primary analyses will compare unadjusted differences between the study groups at each follow-up. Supplementary adjusted analyses, with adjustment for potential covariates (baseline values of relevant outcome, age, sex, use of medication, chronic conditions, falls history and other background variables that show imbalances between the intervention and control groups) will also be conducted.

Missing data: where possible, we will obtain endpoint measures from all withdrawals and include all randomised subjects in the final analysis. For participants who are lost to follow- up, missing data will be handled with multiple imputation. As this approach makes an untestable assumption that data are missing at random (that is, missing data can be predicted from the observed data) [77], we will perform sensitivity analysis to evaluate the effect of potential non-random attrition [78]. Sensitivity analyses will employ simulation and will test a range of scenarios assuming plausible arm-specific differences in outcomes for individuals who were lost to follow-up [79].

## Discussion

Many falls in older people result from an inability to generate sufficient lower limb muscle power to produce an explosive and rapid movement to step quickly when balance is lost. This is confounded further when simultaneously performing concurrent attention-demanding tasks (‘dual-task’ paradigm), such as talking while walking or negotiating traffic or obstacles. Despite the clinical relevance of muscle power and dual-tasking to ‘*real life*’ situations*,* no studies have examined the efficacy of either approach for preventing falls in a large-scale randomised controlled trial. We expect that the results from this trial will guide the development and implementation of future community-based falls prevention programmes that specifically focus on optimising muscle power and function and reducing falls risk under ‘*real-life’* conditions in older adults at risk of falling. If successful, the pragmatic design of the exercise intervention programme could be easily adopted to routine practice. The successful delivery of the intervention within retirement villages is also critical as it overcomes common barriers to exercise participation for older people. Furthermore, the ‘step down’ programme will provide important new information about the efficacy of a less intensive maintenance programme for reducing the risk of falls over an extended period in community-dwelling older adults.

## Trial status

Recruitment is currently underway and a number of participants have commenced the study.

## Abbreviations

1-RM: 1-repetition maximum strength
ADL: Activities of daily living
AEP: Accredited exercise physiologist
AQoL-6D: Assessment of quality of life - 6D scale
BIA: Bioelectrical impedance
CEA: Cost-effectiveness analysis
CHAMPS: Community healthy activities model programme for seniors
CSRT: Choice stepping reaction time
DT-FPT: Dual-task functional power training
DQES v2: Dietary questionnaire for epidemiological studies version 2
ESSA: Exercise and sports science Australia
FES-I: Falls efficacy scale-international
FM: Fat mass
FFM: Fat-free mass
FFQ: Food frequency questionnaire
FSST: Four-square step test
HR-QoL: Health-related quality of life
HV-PRT: High-velocity progressive resistance training
IADL: Instrumental activities of daily living
ICD-10: International classification of diseases, 10th edition
ICER: Incremental cost-effectiveness ratio
MCS: Mental component summary score
NHMRC: National health and medical research council
NIA: National institute for ageing
PCS: Physical component summary score
PKMAS: ProtoKinetics movement analysis software
PRT: Progressive resistance training
QoL: Quality of life
SF-36 v2: Short form 36 version 2
STS: Sit-to-stand test
TUG: Timed up-and-go test
